# Tailoring implementation strategies for evidence-based recommendations using computerised clinical decision support systems: protocol for the development of the GUIDES tools

**DOI:** 10.1186/s13012-016-0393-7

**Published:** 2016-03-05

**Authors:** Stijn Van de Velde, Pavel Roshanov, Tiina Kortteisto, Ilkka Kunnamo, Bert Aertgeerts, Per Olav Vandvik, Signe Flottorp

**Affiliations:** 1 Norwegian Institute of Public Health, Oslo, Norway; 2 McMaster University, Hamilton, Canada; 3 Tampere University Hospital, Tampere, Finland; 4 Duodecim, Scientific Society of Finnish Physicians, Helsinki, Finland; 5 Department of Public Health and Primary Care, Katholieke Universiteit Leuven, Leuven, Belgium; 6 MAGIC Non-Profit Research and Innovation Programme, Norwegian Institute of Public Health, Oslo, Norway

## Abstract

**Background:**

A computerised clinical decision support system (CCDSS) is a technology that uses patient-specific data to provide relevant medical knowledge at the point of care. It is considered to be an important quality improvement intervention, and the implementation of CCDSS is growing substantially. However, the significant investments do not consistently result in value for money due to content, context, system and implementation issues. The Guideline Implementation with Decision Support (GUIDES) project aims to improve the impact of CCDSS through optimised implementation based on high-quality evidence-based recommendations. To achieve this, we will develop tools that address the factors that determine successful CCDSS implementation.

**Methods/design:**

We will develop the GUIDES tools in four steps, using the methods and results of the Tailored Implementation for Chronic Diseases (TICD) project as a starting point: (1) a review of research evidence and frameworks on the determinants of implementing recommendations using CCDSS; (2) a synthesis of a comprehensive framework for the identified determinants; (3) the development of tools for use of the framework and (4) pilot testing the utility of the tools through the development of a tailored CCDSS intervention in Norway, Belgium and Finland. We selected the conservative management of knee osteoarthritis as a prototype condition for the pilot. During the process, the authors will collaborate with an international expert group to provide input and feedback on the tools.

**Discussion:**

This project will provide guidance and tools on methods of identifying implementation determinants and selecting strategies to implement evidence-based recommendations through CCDSS. We will make the GUIDES tools available to CCDSS developers, implementers, researchers, funders, clinicians, managers, educators, and policymakers internationally. The tools and recommendations will be generic, which makes them scalable to a large spectrum of conditions. Ultimately, the better implementation of CCDSS may lead to better-informed decisions and improved care and patient outcomes for a wide range of conditions.

**Protocol registration:**

PROSPERO, CRD42016033738

**Electronic supplementary material:**

The online version of this article (doi:10.1186/s13012-016-0393-7) contains supplementary material, which is available to authorized users.

## Background

Application of the best research evidence in clinical practice can improve the quality and safety of health care. Successfully translating evidence into practice requires that clinicians are aware of the evidence, agree with it, are confident about delivering the intervention and adhere to it in appropriate situations [1]. Furthermore, patients should agree and adhere to the treatment [1]. When there is more than one reasonable healthcare option, decision-making involves weighing the benefits and harms of the options, often with scientific uncertainty, and the preferences of the patients. Unfortunately, there is often a gap between the recommended care and the care that patients receive, and patient adherence to appropriate care can be poor [1]. Furthermore, some healthcare interventions may not be needed or may even be harmful. Finally, expenses can decrease if care options are chosen according to their comparative cost-effectiveness [2].

A computerised clinical decision support system (CCDSS) is an information technology to aid clinicians and patients in making healthcare decisions, based on patient-specific data [3]. This is a broad term, and CCDSS comes in many types and functions [4]. It can be Internet-based, installed on a local personal computer or a networked electronic health record or function on a handheld device. The computer-generated decision support can be provided on screen or on paper. By linking healthcare providers with relevant guidelines at the point of care, CCDSS can improve the adherence to evidence-based recommendations [5]. CCDSS can also empower and motivate patients by providing patient-directed advice and improve disease self-management [6, 7]. Further, CCDSS could facilitate shared decision-making by healthcare providers and patients together by providing decision aids [8, 9].

Hopeful governments have made substantial investments in healthcare information technology with decision support capabilities [10, 11]. It has become apparent, however, that the required investment is greater than initially planned and the results of many initiatives fall short of expectation [12, 13]. Systematic reviews of CCDSS have reported modest improvements in the healthcare process [14, 15]. Even the newest generation of CCDSS, touted as evidence-based and fully integrated in the electronic health record, deliver only modest reductions in morbidity [16].

Despite popular claims, the mere integration of CCDSS in electronic health records is not sufficient to obtain clinical benefit. Some studies estimate that alerts are ignored in 49 to 96 % of the cases [17]. Even though CCDSS can potentially improve the quality of care, it also holds the risk of new types of adverse effects, sometimes leading to patient harm [18, 19]. Unintended consequences of CCDSS come in various categories, with a report mentioning 47 types. Alert fatigue is a well-known example; less well known is automation bias where users tend to over-rely on computer output [20, 21]. Over 380 examples have been described for computerised provider order-entry systems, which are often integrated with CCDSS [22]. Furthermore, recommendations provided by CCDSS are not always up-to-date or evidence-based and failing to meet standards for trustworthiness, well agreed upon in the guideline community [23]. The benefits for patients remain uncertain if these problems are not addressed.

The Tailored Implementation for Chronic Diseases (TICD) project is developed of methods to tailor knowledge implementation interventions in chronic illness care [24] consistent with evidence of the benefits of tailored implementation [25]. In tailored implementation, the strategies for knowledge translation are based on an assessment of determinants of healthcare practice to achieve desired changes [25]. The TICD project, however, did not address the specifics of CCDSS implementation.

Current CCDSS implementations are often only superficially tailored to local context and do not always involve the users, neither the patients nor the health professional [26, 27]. Although multiple determinants of successful development and use of CCDSS have been proposed [5, 28, 29], some have been contested again [12]. As stated in a recent editorial by the lead author of a comprehensive systematic review, we are only beginning to understand what factors make the implementation of decision support effective and it might be necessary to chart a new course [26]. Caution is needed since the implementation of CCDSS systems is growing substantially [30].

Therefore, with the Guideline Implementation with Decision Support project (GUIDES), we aim to improve the impact of CCDSS through optimised implementation, based on high-quality and trustworthy decision support content [31]. Here, we describe our methods to (1) investigate the factors that determine successful implementation strategies for evidence-based recommendations with CCDSS, (2) develop tools such as checklists, worksheets, and practical recommendations to address these factors and (3) validate the utility of these tools through the development of a tailored implementation strategy.

## Methods/design

**Fig. 1:**
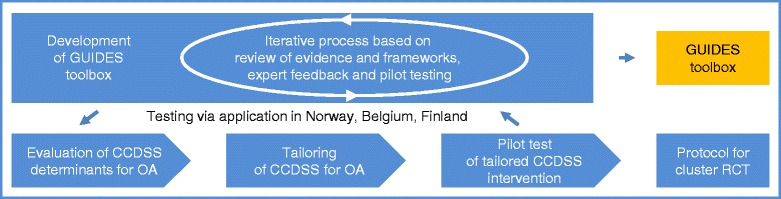
Schematic overview of the GUIDES project. Legend: *CCDSS* Computerised Clinical Decision Support System, *OA* osteoarthritis

We will develop the GUIDES tools in four steps, using the methods and results of the TICD project as a starting point:

1. A review of research evidence and frameworks on the determinants of implementing recommendations using CCDSS
2. A synthesis of a comprehensive framework for the identified determinants
3. The development of tools for use of the framework and
4. Pilot testing the utility of the tools through the development of a tailored CCDSS intervention. Finally, the results from the actions described here will inform the design of a large-cluster randomised controlled trial to evaluate the effectiveness of the tailored CCDSS and for identifying optimal implementation strategies in computerised decision support. Figure 1 provides a schematic overview of the GUIDES project.

The GUIDES author group includes experts from different countries (Belgium, Canada, Finland and Norway) with a strong commitment to evidence-based medicine and broad expertise related to the development, implementation and evaluation of CCDSS [12, 24, 32–36]. Two authors (IK, POV) are also involved in the development of systems for decision support, which potentially could create a conflict of interest. If a conflict would arise for specific implementation determinants, these experts will not participate in the discussion on how to develop guidance for this item [37]. The authors will also collaborate with an international expert group to provide input and feedback on the tools.

### Review of research evidence and frameworks

Two researchers (SVDV, SF) will use systematic methods to review frameworks, systematic reviews, process evaluations and qualitative evidence pertaining to factors for successful CCDSS implementation. The research questions are: (1) Which factors contribute to the successful implementation of CCDSS; (2) Which frameworks are being used to organise these factors and (3) What is the evidence for the impact of these factors?

We focus on the implementation of evidence-based recommendations using CCDSS, but we also include research evidence and frameworks for the implementation of CCDSS in general. We consider the different types of CCDSS with any objective (e.g. diagnosis, treatment, test ordering, screening), in any healthcare setting and directed at healthcare professionals and/or patients. From a holistic point of view, we also include papers on the implementation of health information systems in general, if CCDSS is included in the scope. We define success as any desired change in the effectiveness, safety, efficiency, responsiveness, or equity of health services, based upon the outcomes as proposed by the Cochrane Effective Practice and Organisation of Care (EPOC) review group [38, 39]. If such data is available, we also consider success as reduction in costs or more efficient use of resources. We exclude papers about sending reminder messages for attendance at upcoming healthcare appointments, studies on health information systems that only target patients or studies on Internet-based treatment of specific conditions.

We define a framework as any overview or classification of determinants of the successful implementation of CCDSS. Some known examples of general frameworks include the Five Rights model or the ten commandments for effective CCDSS [5, 29]. Frameworks will be included if they allow the extraction of data that can be formulated as determinants for the implementation of CCDSS. We exclude frameworks if they are too specific (e.g. only locally applicable or limited to a specific CCDSS product) or too broad to operationalise them into a checklist.

The screening for systematic reviews will focus on reviews that allow the identification of factors related to successful implementation of CCDSS based on randomised controlled trials. Given that multiple systematic reviews are available, we will select the most recent reviews, those with a broad scope that includes multiple settings, clinical domains or CCDSS functions and those of the best methodological quality. When excluding reviews, we will check that no important information on potential factors is lost.

Further, we will identify process evaluations or qualitative studies that followed trials on the effectiveness of CCDSS [40, 41]. Such studies can provide valuable insights in the intended or unintended effects of an intervention. We define qualitative studies as studies that used collected data using focus groups, individual interviews, observation and document analysis, and that used qualitative methods to analyse the data [42, 43]. If systematic reviews of qualitative studies or process evaluations are available, these are also included.

After an initial screening for factorial trials on CCDSS success features, we decided to also conduct a systematic review of randomised trials that compare the impact of different CCDSS determinants. This also includes trials on the effect of adjacent interventions (e.g. academic detailing) to increase the success of CCDSS. A protocol for this systematic review is registered in the PROSPERO database (CRD42016033738).

The search strategy is described in the Appendix. Our own files and the reference lists of relevant papers will supplement this search. To select the studies, the two reviewers will screen titles and abstracts of all the retrieved references. One author (SVDV) will then screen full texts and further exclude irrelevant studies. Any disagreements will be resolved by discussion with a third person (POV). We do not apply any language restrictions.

### Synthesis of results in a comprehensive framework

The reviewers will independently assess the selected frameworks according to desirable framework attributes as defined in the TICD project [44].

One reviewer (SVDV) will extract the identified domains and determinants from each selected paper and will use the list of identified determinants and domains to compose a draft framework. To construct this, we will expand on the TICD checklist [44]. When the specificity of CCDSS requires this, we will adapt existing items, add new concepts or exclude irrelevant items. For every included factor, we foresee a short explanation. SF will quality check the new aggregated list by comparing it with the TICD checklist and the list of factors identified in the review.

If systematic reviews or reviews of qualitative studies provide evidence for the importance of a specific determinant, we will indicate the confidence we have in the evidence as high, moderate or low. Depending on the type of evidence, we will assess this by means of the GRADE or CerQual approach [42, 45]. To evaluate the quality, two reviewers will independently apply the ROBIS tool for systematic reviews and the CerQual tool for reviews of qualitative evidence [42, 46].

Next, we will invite experts within the field and CCDSS developers or implementers to evaluate the preliminary framework. To compile the list of people, we will include the corresponding authors of every relevant paper found in the review phase and combine this with the suggestions by the members of the GUIDES project. It is our goal to include persons across different country settings. Experts will be requested to declare potential conflicts of interests. Every participating expert will receive the preliminary framework and the first part of a structured feedback form with desirable framework attributes (see Additional file 1). The feedback form has been tested previously during the TICD project. We will not use formal consensus methods during this consultation of the expert group. SVDV will summarise the scores per question and will make a thematic summary of open-ended comments or suggestions. SF will verify the accuracy of this procedure. The group of authors will then discuss the feedback and agree in consensus on the revisions that should be made.

Next, we will send the revised version to the expert group with the request to score independently each item in the framework on the question ‘How important is this determinant for the success of a CCDSS intervention’ by means of a Likert scale from 1 (not important) to 5 (very important). Then, for each item, we will calculate the median score, range and percentage of ‘4’ and ‘5’ scores.

### Development of the GUIDES tools

Based on the new framework, the existing TICD worksheets, discussion among the authors and feedback from key experts, we plan to develop tools to support the tailoring of implementation strategies for recommendations with CCDSS. We will also integrate the findings from the DECIDE project, which develops and evaluates communication strategies to support evidence informed decisions [47].

The tools will contain an overview of CCDSS implementation success factors and best practices, checklists and worksheets regarding the identification of implementation determinants and the matching to implementation interventions. They will also contain practical recommendations on planning and running tailored CCDSS implementation interventions.

Each tool will include definitions and explanations on how to use it combined with examples. To ensure usability and usefulness of the tools, we will consider its comprehensiveness, consistency of framing, and usability. The group of experts will receive another invitation for feedback, this time on the tools that have been developed using part 2 of the feedback form (Additional file 1).

### Pilot testing of the GUIDES tools

The tools will be tested through the development of a tailored implementation strategy with CCDSS for patients with knee osteoarthritis. From many potentially relevant conditions, we have chosen knee osteoarthritis as a prototype because it is associated with considerable disability in the general population, it is a high economic burden, causes a major public health problem and quality indicator pass rates are remarkably low [48–50]. The clinical information used in the CCDSS will come from high-quality clinical practice guidelines, systematic reviews and patient decision aids on knee osteoarthritis [51, 52].

We submitted the protocol for this pilot study as a separate publication in *Implementation Science*. In short, we will organise focus groups in Norway, Belgium and Finland to inform the development of a tailored CCDSS implementation strategy for knee osteoarthritis recommendations. We will test decision support both for situations related to strong and weak (or conditional) recommendations and for comorbidity-related issues. The experience from these applications will be used to fine-tune the tools.

To get multiple perspectives on the use of the GUIDES tools, we also plan to invite CCDSS developers and implementers to a workshop [53]. We will collect their feedback and discuss within the author group if any adjustments to the tools are needed.

Further, we will use the GUIDES framework in a systematic review of trials on CCDSS success features (PROSPERO database registration number CRD42016033738). During the data extraction process, pairs of reviewers will independently code the identified trials using the determinants that are listed in the GUIDES framework. We will calculate inter-rater agreement and record the frequency of coding [54].

## Discussion

It is clear that substantial work is necessary to realise the full potential of CCDSS, and guidance for the optimal design and implementation of CCDSS is urgently required [26]. This project will provide guidance and tools on methods of identifying implementation determinants and selecting strategies to implement evidence-based recommendations through CCDSS. We will make the GUIDES tools available to CCDSS developers, implementers, researchers, funders, clinicians, managers, educators and policymakers internationally. The tools and recommendations will be generic which makes them scalable to a large spectrum of conditions. Due to time and budgetary constraints, we can only pilot the GUIDES tools for one clinical condition. This is a limitation of the project since implementation determinants might differ between clinical topics.

Having patient information available in the electronic health record in a structured/coded format is considered to be important for the implementation of CCDSS [55]. This allows healthcare professionals to record the patient characteristics and makes it possible for computers to process the data. However, the design of clinical documentation tools to bridge the gap between daily medical data and international nomenclatures and classifications remains a challenge. Furthermore, these tools need to be adopted by the healthcare providers and to date, significant amounts of patient data are not available in a structured/coded format [56]. Overcoming this barrier is obviously a challenge that is wider than the objectives and the time span of this project.

## Appendix

### Search strategy

#### Search for frameworks and systematic reviews

We searched the following:

MEDLINE (Ovid) 1946 to present

CINAHL (EBSCO) 1981 to present

Science Citation Index & Social Sciences Citation Index (ISI Web of Science) 1955 to present

Epistemonikos

PsycInfo (Ovid) 1987 to present

Search formula for MEDLINE:

1. decision support systems, clinical/
2. decision making, computer-assisted/
3. diagnosis, computer-assisted/
4. therapy, computer-assisted/
5. Reminder Systems/
6. Information Systems/
7. Health Information Systems/
8. reminder systems.sh.
9. decision support.tw.
10. or/1-9
11. Classification/
12. Models, Organizational/
13. st.fs.
14. cl.fs.
15. or/11-14
16. (barrier? or obstacle? or impediment? or hinder* or enabler? or facilitator? or moderator? or mediator? or driver? or modifier? or determinant? or feature? or factor? or theme? or element? or domain?).tw.
17. 15 and 16
18. ((taxonomy or taxonomies or ontology or ontologies or framework? or frame work? or classify or classification? or theory or theories or theoretical or standard? or plan) adj4 (barrier? or obstacle? or impediment? or hinder* or enabler? or facilitator? or moderator? or mediator? or driver? or modifier?or determinant? or feature? or factor? or theme? or element? or domain?)).tw.
19. 17 or 18
20. MEDLINE.tw.
21. systematic review.tw.
22. meta-analysis.pt.
23. intervention$.ti
24. or/20-23
25. (barrier? or obstacle? or impediment? or hinder* or enabler? or facilitator? or moderator? or mediator? or driver? or modifier? or determinant? or feature? or factor? or theme? or element? or domain?).tw.
26. 24 and 25
27. 19 or 26
28. 10 and 27

#### Search for process evaluations or qualitative evidence associated to trials

We searched the following:

MEDLINE (Ovid) 1946 to present

CINAHL (EBSCO) 1981 to present

Science Citation Index & Social Sciences Citation Index (ISI Web of Science) 1955 to present

PsycInfo (Ovid) 1987 to present

Cochrane Central Register of Controlled Trials: Issue 8 of 12, August 2015

Epistemonikos: We will screen the references to trials identified through an automatic evidence matrix for the review by Roshanov et al. This matrix provides all the systematic reviews that share at least one study along with the references to the included studies. We only select those reviews with a focus on CCDSS or health information systems.

Search formula for MEDLINE:

1. decision support systems, clinical/
2. decision making, computer-assisted/
3. diagnosis, computer-assisted/
4. therapy, computer-assisted/
5. Reminder Systems/
6. Information Systems/
7. Health Information Systems
8. reminder systems.sh.
9. decision support.tw.
10. or/1-9
11. factorial.mp.
12. 2 by 2.mp.
13. 2 × 2.mp.
14. 11 or 12 or 13
15. randomised controlled trial.pt.
16. controlled clinical trial.pt.
17. randomized.ab.
18. placebo.ab.
19. drug therapy.fs.
20. randomly.ab.
21. trial.ab.
22. groups.ab.
23. or/15-22
24. exp animals/not humans.sh.
25. 23 not 24
26. 14 and 25
27. ((("semi-structured" or semistructured or unstructured or informal or "in-depth" or indepth or "face-to-face" or structured or guide) adj3 (interview* or discussion* or questionnaire*)) or (focus group* or qualitative or ethnograph* or fieldwork or "field work" or "key informant")).ti,ab. or interviews as topic/or focus groups/or narration/or qualitative research/
28. 25 and 27
29. "Process Assessment (Health Care)"/
30. exp Program Evaluation/
31. (process adj3 (evaluat* or assess*)).ti,ab.
32. or/29-31
33. 25 and 32
34. 26 or 28 or 33
35. 10 and 34

## Additional file

Additional file 1: Structured feedback form with desirable framework attributes to evaluate the preliminary GUIDES framework.
