# Supplementary material for: Tailoring implementation strategies for evidence-based recommendations using computerised clinical decision support systems: protocol for the development of the GUIDES tools
Source: Implement Sci. 2016 Mar 5;11:29. doi: 10.1186/s13012-016-0393-7 (PMC4779557; doi:10.1186/s13012-016-0393-7)
Supplement: Additional file 1: — Structured feedback form with desirable framework attributes to evaluate the preliminary GUIDES framework. [file 13012_2016_393_MOESM1_ESM.doc]

## Appendix 2: Structured feedback form

| **Name(s):** | | |
| --- | --- | --- |
| **Date:** |  |  |

| **Part 1: Feedback on the framework** | | |
| --- | --- | --- |
|  |  | **Comments (including explanations of perceived problems and suggestions for improvements)** |
| **Comprehensiveness**   1. **Are potentially important factors missing from the framework?** | | Yes | Uncertain | No | | --- | --- | --- | | 🞎 | 🞎 | 🞎 | |  |
| **Relevance**   1. **Are factors included in the framework that should not be?** | | No | Uncertain | Yes | | --- | --- | --- | | 🞎 | 🞎 | 🞎 | |  |
| **Applicability**   1. **Is the framework applicable across different settings (e.g. primary and secondary care) and different types of practices (including prevention, diagnosis and treatment for chronic and non-chronic conditions)?** | | Yes | Uncertain | No | | --- | --- | --- | | 🞎 | 🞎 | 🞎 | |  |
| **Simplicity**   1. **Is the framework more complicated than necessary?** | | No | Uncertain | Yes | | --- | --- | --- | | 🞎 | 🞎 | 🞎 | |  |
| **Logic**   1. **Is the framework organised in a logical way that is easy to understand?** | | Yes | Uncertain | No | | --- | --- | --- | | 🞎 | 🞎 | 🞎 | |  |
| **Clarity**   1. **Are the factors and domains (groups of factors) labelled and explained in a way that is easy to understand?** | | Yes | Uncertain | No | | --- | --- | --- | | 🞎 | 🞎 | 🞎 | |  |
| **Overall assessment**   1. **Overall, is the framework adequate to be used to identify and prioritise determinants of practice?** | | Yes | Partially | No | | --- | --- | --- | | 🞎 | 🞎 | 🞎 |   See explanation below. |  |
| 1. **Would you use this framework?** | | Yes | Partially | No | | --- | --- | --- | | 🞎 | 🞎 | 🞎 | | |
| **Strengths**   1. What things do you like about the framework? |  | |
| **Weaknesses**   1. What things don’t you like about the framework and what suggestions do you have for improving them? |  | |
| **Anything else**   1. Please include any other comments you have regarding the framework. |  | |

**Overall assessment**

Yes = Could be used as is with little or no modification

Partially = Needs some modification or further development

No = Not adequate

| **Part 2: Feedback on the GUIDES tools** | | |
| --- | --- | --- |
|  |  | **Comments (including explanations of perceived problems and suggestions for improvements)** |
| **Usability**   1. **Would it be easy for CCDSS implementation researchers to use the tools?** | | Yes | Uncertain | No | | --- | --- | --- | | 🞎 | 🞎 | 🞎 | |  |
| 1. **Would it be easy for people who are not CCDSS implementation researchers to use the tools?** | | Yes | Uncertain | No | | --- | --- | --- | | 🞎 | 🞎 | 🞎 | |  |
| **Suitability**   1. **Are the tools suitable for helping people to identify and prioritise determinants of successful decision support that should be considered when designing CCDSS implementation strategies?** | | Yes | Uncertain | No | | --- | --- | --- | | 🞎 | 🞎 | 🞎 | |  |
| **Usefulness**   1. **Are the tools likely to be useful to people designing implementation strategies?** | | Yes | Uncertain | No | | --- | --- | --- | | 🞎 | 🞎 | 🞎 | |  |
| 1. **Is the checklist likely to be useful for reporting determinants of successful decision support in research reports?** | | Yes | Uncertain | No | | --- | --- | --- | | 🞎 | 🞎 | 🞎 | |  |
| **Overall assessment**   1. **Overall, are the tools adequate to be used to identify and prioritise determinants of successful decision support?** | | Yes | Partially | No | | --- | --- | --- | | 🞎 | 🞎 | 🞎 |   See explanation below. |  |
| 1. **Would you use this framework?** | | Yes | Partially | No | | --- | --- | --- | | 🞎 | 🞎 | 🞎 | |  |
| **Strengths**   1. What do you like about the tools? |  | |
| **Weaknesses**   1. What don’t you like about the tools and what suggestions do you have for improving them? |  | |
| **Anything else**   1. Please include any other comments you have regarding the tools. |  | |
